# The Self-Information Weighting-Based Node Importance Ranking Method for Graph Data

**DOI:** 10.3390/e24101471

**Published:** 2022-10-15

**Authors:** Shihu Liu, Haiyan Gao

**Affiliations:** School of Mathematics and Computer Sciences, Yunnan Minzu University, Kunming 650504, China

**Keywords:** graph data, information entropy, node importance ranking, self-information weighting

## Abstract

Due to their wide application in many disciplines, how to make an efficient ranking for nodes, especially for nodes in graph data, has aroused lots of attention. To overcome the shortcoming that most traditional ranking methods only consider the mutual influence between nodes but ignore the influence of edges, this paper proposes a self-information weighting-based method to rank all nodes in graph data. In the first place, the graph data are weighted by regarding the self-information of edges in terms of node degree. On this base, the information entropy of nodes is constructed to measure the importance of each node and in which case all nodes can be ranked. To verify the effectiveness of this proposed ranking method, we compare it with six existing methods on nine real-world datasets. The experimental results show that our method performs well on all of these nine datasets, especially for datasets with more nodes.

## 1. Introduction

Node importance ranking [1] aims to construct a suitable score function for all nodes, in which case all nodes can be ranked with the help of this score function.Especially in recent years, with the popularity of graph data, the problem of node importance ranking for graph data has been widely studied and applied in many fields, such as blocking rumors [2,3], disease detection [4,5], information transmission [6,7], and so on.

To date, for the problem of node importance ranking, the methods of constructing score function can be roughly classified into three categories, which are local-information-based score functions [8,9], global-information-based score functions [10,11] and node-position-based score functions [12,13]. The local-information-based score functions mainly consider the local topology information of node itself and neighbors. Although they have low complexity, the accuracy of the rank result is also low. The global-information-based score functions usually need to traverse the entire graph data, so they might have the expensive time costs and cannot be directly applied to large-scale graph data. The node-position-based score functions are relatively rare, because these methods usually assign the same score to a large number of nodes and cannot accurately identify their importance.

Bearing what was discussed above in mind, plenty of methods have been proposed and investigated carefully. For instance, the degree centrality [14] method constructed the simplest local-information-based score function. It defined the importance of nodes as the number of neighbors, which reflected the direct influence of a node on others. Zhang et al. [15] analogized the problem of node importance ranking to the voting process based on the degree of neighbors. The eigenvector centrality [16] method determines the importance of nodes by taking the eigenvalues and eigenvectors of adjacency matrix into consideration, which constructs a global-information-based score function. Fu et al. [17] constructed the two-step framework that combines the global information and local topology features to identify influential nodes. The closeness centrality [18] method quantifies the importance of nodes by calculating the average distance from one node to all other nodes. The betweenness centrality [19] method characterizes node importance as the number of shortest paths through the node. The more times a node acts as the bridge, the more important it is. The K-shell decomposition centrality [20] method recursively deletes nodes in the outer layer of the graph data. It considers that the nodes at the core of graph data have strong influence. The PageRank method [21] was applied in the Google search engine, and considers each web page as a node and hyperlinks between pages as edges. The importance of node in PageRank method depends on the importance of other nodes pointed to this node. Wang et al. [22] proposed a label propagation algorithm based on the similarity to identify the influential node. The problem of node importance ranking is regarded as a multi-attribute decision making problem in reference [23], which can take many factors that affect the importance of nodes into account.

Besides the above mentioned, the theory of entropy has been used by many researchers to deal with the problem of node importance ranking [24,25,26]. For example, Guo et al. [27] proposed the VoteRank algorithm, which introduced information entropy as the influence of node on its neighbors. Zareie et al. [28] used information entropy while considering the degree distribution of first-order neighbors and second-order neighbors of nodes. Based on the hypothesis that the removal of a more important node is likely to cause more structural variation, entropy variation [29] is proposed to study the problem of node importance ranking. The local structure entropy approach [30], proposed by Lei et al., comprehensively considers the relationship between a node’s Tsallis entropy and its neighbors. Fei et al. [31] proposed a novel method to identify influential nodes using relative entropy and TOPSIS method, which combines the advantages of existing centrality measures.

Although the accuracy of rank results can be improved with the help of entropy, most of these methods only consider the mutual influence between nodes and ignore the influence of edges that directly connected to the node itself [32,33,34]. Certainly, as the important component of graph data, the information contained in the edges can make a huge influence for the final ranking [35]. Therefore, how to measure the amount of information contained in the edges and make full use of them is vital.

Inspired by the studies mentioned above, in this paper, we will still study the problem of node importance ranking for graph data. However, here we pay attention to the edge and propose a self-information weighting-based node importance ranking method. In summary, this paper makes the following contributions:

- The graph data are weighted by regarding the self-information of edges in terms of the node degree.
- The information entropy of nodes is constructed to measure the importance of each node. What is more, the rank result can be obtained according to the value of the information entropy.
- Nine real-world datasets are used to show the validity of the self-information weighting-based node importance ranking method for graph data. The experimental results manifest that our method has great advantage in terms of monotonicity, node distribution and accuracy.

The remainder of this paper is organized as follows. Section 2 makes a brief review of some basic knowledge. Section 3 introduces the proposed node importance ranking method, i.e., the self-information weighting-based node importance ranking method. Section 4 is composed of three parts, which are experimental platform, datasets description and evaluation criteria. Section 5 shows the detailed comparison between the proposed node importance ranking method and some existing ranking methods on nine real-world datasets. Section 6 concludes this paper and also makes a possible direction for future research.

## 2. Preliminaries

In this section, we propose some basic concepts that are closely related to the work of this article, such as graph data and the benchmark methods of how to rank the nodes. For more detailed description, one can refer to the Refs. [36,37,38,39].

### 2.1. Graph Data

Mathematically, the so-called graph data can be expressed as a tuple G=(V,E), where

- $V=\left\{v_{1},v_{2},\cdots ,v_{n}\right\}$ is the collection of nodes and *n* represents the number of nodes.
- $E=\left\{\left(v_{i},v_{j}\right)|v_{i},v_{j}\in V\right\}$ is the collection of edges, in which case $(v_{i},v_{j})\in E$ means that there is an edge between nodes $v_{i}$ and $v_{j}$. As that of *V*, we apply *m*, i.e., |E|=m, to denote the number of edges.

Without loss of generality, in this paper we adhere to the hypothesis that the graph data G=(V,E) is an undirected and unweighted graph data. In other words, $\left(v_{i},v_{j}\right)=\left(v_{j},v_{i}\right)$ for any $v_{i},v_{j}\in V$. In addition, adjacency matrix of graph data G=(V,E) can be expressed as a matrix

$$A=\left(\begin{array}{cccc} a_{11} & a_{12} & \cdots & a_{1n}\\ a_{21} & a_{22} & \cdots & a_{2n}\\ \vdots & \vdots & \ddots & \vdots \\ a_{n1} & a_{n2} & \cdots & a_{nn} \end{array}\right),$$

where $a_{ij}$ represents the connectivity between nodes $v_{i},v_{j}\in V$, for i,j=1,2,⋯,n. Obviously, $a_{ij}=1$ if and only if $(v_{i},v_{j})\in E$, otherwise $a_{ij}=0$.

### 2.2. Benchmark Methods for Node Importance Ranking

The key step of node importance ranking is to construct a suitable score function for all nodes, in which case all nodes can be ranked with the help of this proposed score function.At present, the existing methods of constructing score function can be divided into three categories: the local-information-based score function, the global-information-based score function and the node-position-based score function.

#### 2.2.1. The Local-Information-Based Score Function

The degree centrality method, abbreviated to DC for convenience, takes the number of neighbor nodes into account to quantify the importance of the node, and the mathematical expression of it can be expressed as

(1) $$DC\left(v_{i}\right)=\sum_{j=1}^{n}a_{ij}.$$

The mutual information method, abbreviated to MI for convenience, defines the mutual information between any two connected nodes as

(2) $$I\left(v_{i},v_{j}\right)=\left\{\begin{array}{cc} ln\left(\frac{d_{i}}{d_{j}}\right) & \;\;a_{ij}=1\\ \; & \;,\\ 0 & \;\;a_{ij}=0 \end{array}\right.$$

where $d_{i}=\sum_{j=1}^{n}a_{ij}$ is the degree of node $v_{i}$. On this base, the amount of information of $v_{i}$ can be defined as

(3) $$MI\left(v_{i}\right)=\sum_{v_{j}\in N\left(v_{i}\right)}I\left(v_{i},v_{j}\right),$$

where $N\left(v_{i}\right)=\left\{v_{j}|\left(v_{i},v_{j}\right)\in E\right\}$ is the set of neighbors of node $v_{i}$.

#### 2.2.2. The Global Information Based Score Function

The closeness centrality method, abbreviated to CC for convenience, defines the importance of nodes as the reciprocal of the average length from one node to all other nodes in graph datum G=(V,E). The corresponding computing formula is

(4) $$CC\left(v_{i}\right)=\frac{n-1}{\sum_{i\neq j}d\left(i,j\right)},$$

where d(i,j) shows the length of the shortest path from node $v_{i}$ to $v_{j}$. If there is no path from $v_{i}$ to $v_{j}$, then d(i,j)=0.

The eigenvector centrality method, abbreviated to EC for convenience, determines the importance of nodes by taking the eigenvalues and eigenvectors of *A*, the adjacency matrix of G=(V,E), into consideration. The calculation formula is defined as

(5) $$EC\left(v_{i}\right)=\lambda^{-1}\sum_{j=1}^{n}a_{ij}e_{j},$$

where λ is the largest eigenvalue, and $e_{j}$ for j=1,2,⋯,n is the *j*th eigenvector of *A*.

#### 2.2.3. The Node Position Based Score Function

The K-shell decomposition method, abbreviated to KS for convenience, evaluates the importance of nodes by sequentially removing nodes in the outer layer of the graph data. The main principle of it is to sign the node with original degree at first, nodes with degree 1 are removed, and this process continues until there are no nodes with degree 1 in the graph data. The importance of all these removed nodes is labeled as 1. Next, nodes with degree 2 are removed. The process continues until there are no nodes with degree less than or equal to 2 in the graph data. Similarly, the importance of these removed nodes is labeled as 2. In addition, nodes with degree 3, 4, ⋯, are removed and labeled until all nodes are completed.

The improved K-shell decomposition method, abbreviated to IKS for convenience, only removes nodes with the lowest degree in the graph data each time, which is the biggest difference between the IKS method and KS method. That is to say, the selection of nodes to remove each time is not necessarily in an increasing sequence of degrees, i.e., 1, 2, ⋯. For example, if all nodes with degree 2 in the graph data are removed in the last iteration and their importance is labeled as 2, but nodes with degree 1 appear in the rest of graph data, these nodes will be removed in the next iteration, and their importance will be labeled as 3. This process continues until all nodes are completed.

## 3. Proposed Method

As can be seen from foregoing discussion, most traditional node importance ranking methods only consider the mutual influence between nodes, while ignoring the influence of edges that directly connected to the node itself. For example, the DC method simply regards the number of neighbors as the importance of nodes. In fact, since each neighbor node has different local topology information, their contributions are not equal. Certainly, the DC method does not distinguish between the contributions of different neighbor nodes, which will lead to unsatisfactory rank results. Bearing this in mind, in what follows, we construct a new method to rank the nodes in graph data G=(V,E). Distinguished from the existing ranking methods, the proposed node ranking method will start from the perspective of edges. Herein, the self-information is regarded as the weight of edges, and it turns an unweighted graph datum into a weighted graph datum. The contribution of neighbor nodes can be distinguished by using different weight values of edges. In this case, the score function used to measure the importance of each node is determined by considering the information entropy of nodes.

### 3.1. Edge Weight Construction in Terms of Self-Information

The self-information proposed by Shannon [40] is usually used to measure the amount of information of a event. Given that $X=\left\{x_{1},x_{2},\cdots ,x_{n}\right\}$ is a discrete random variable and its probability distribution is expressed as $P=\left\{p_{1},p_{2},\cdots ,p_{n}\right\}$, then the self-information of each event $x_{i}\in X$ can be expressed as

(6) $$W\left(x_{i}\right)=-log_{2}p_{i}.$$

The self-information indicates that the amount of information contained in a basic event is inversely proportional with its probability of occurrence. In other words, frequent events usually contain less information. Conversely, events that occur less often contain huge amounts of information. Taking node $v_{i}$ for example, the nodes that have edges connected to $v_{i}$ are much smaller than those without edges connected to $v_{i}$ in the whole network. According to the definition of self-information, these edges contain more valuable information. Therefore, we construct the weight of these edges with the help of the self-information.

Certainly, the degree of any two nodes, taking $v_{i},v_{j}\in V$ for example, can be applied to depict the information of corresponding edge $(v_{i},v_{j})\in E$ to some extent. Even more, the amount of information obtained from this can be used to describe the weight of corresponding edge. Bearing what was discussed above in mind, we have that the probability corresponding to any $(v_{i},v_{j})\in E$ can be defined as

(7) $$P\left(v_{i},v_{j}\right)=\frac{1}{d_{i}d_{j}},$$

where $d_{i}$ is the degree of $v_{i}$, the same as that of $d_{j}$.

To this, the self-information of edge $\left(v_{i},v_{j}\right)$ is equivalent to its weight, in which case it can be calculated by the following equation

(8) $$\begin{array}{cc} W(v_{i},v_{j}) & =-log_{2}P(v_{i},v_{j})\\ & =log_{2}\left(d_{i}d_{j}\right). \end{array}$$

Obviously, we can find that for the graph data G=(V,E), it is easy to obtain the equation

(9) $$W\left(v_{i},v_{j}\right)=W\left(v_{j},v_{i}\right).$$

The reason of it is that on one hand, the Equation (9) can be obtained from Equation (8). On the other hand, as G=(V,E) is an undirected graph data, $(v_{i},v_{j})\in E$ if and only if $(v_{j},v_{i})\in E$, and both of edges $\left(v_{i},v_{j}\right)$ and $\left(v_{j},v_{i}\right)$ should have the same self-information.

### 3.2. Node Importance Induced by Information Entropy

Given that *X* is a random variable and its corresponding probability distribution is $\vec{P}=\left(p_{1},p_{2},\cdots ,p_{n}\right)$, if we let $W\left(X\right)={\left(W\left(x_{1}\right),W\left(x_{2}\right),\cdots ,W\left(x_{n}\right)\right)}^{T}$, then we have the following equation based on Equation (6).

(10) $$\begin{array}{cc} \vec{P}\cdot W\left(X\right) & =\left(p_{1},p_{2},\cdots ,p_{n}\right)\left(\begin{array}{c} W(x_{1})\\ W(x_{2})\\ \vdots \\ W(x_{n}) \end{array}\right)\\ & =-\sum_{i=1}^{n}p_{i}log_{2}p_{i}. \end{array}$$

Up to now, $\vec{P}\cdot W\left(X\right)$, abbreviated to E(X) for convenience, can be regarded as the expected value of self-information. According to Equation (6), the negative log of probability represents the amount of information contained in a basic event, i.e., the self-information. The expected value of the amount of information contained in all basic events is called information entropy. In other words, it can be applied to quantify the amount of information contained in the random variable *X*. Herein, we use information entropy to quantify the importance of nodes mainly because of the special properties of information entropy. Following the ideology of the above Equation (10), the properties of information entropy are listed as follows.

**Property** **1.** *Given that X is a random variable and its corresponding probability distribution is $\vec{P}$, then we have that E(X) reaches the maximum when $\vec{P}$ is an uniform distribution.*

**Proof.**  Obviously, for all $p_{i}\in \vec{P}$, one has that the following constraint

(11) $$\sum_{i=1}^{n}p_{i}=1$$

is correct. With Equations (10) and (11), we construct the Lagrange function as

(12) $$\begin{array}{cc} L(p_{1},p_{2},\cdots ,p_{n},\lambda ) & =E\left(X\right)+\lambda \left(\sum_{i=1}^{n}p_{i}-1\right)\\ & =-\sum_{i=1}^{n}p_{i}log_{2}p_{i}+\lambda \left(\sum_{i=1}^{n}p_{i}-1\right). \end{array}$$

By considering the partial derivative of each variable $p_{i}$, then let all of them be equal to zero. With this operation, one can have that

(13) $$p_{i}=e^{\lambda -1}\;\left(i=1,2,\cdots ,n\right).$$

With the help of Equations (11) and (13), the following result

(14) $$p_{i}=\frac{1}{n}\;\left(i=1,2,\cdots ,n\right)$$

comes naturally. Once *n* is fixed, the probability distribution $\vec{P}$ will be an uniform distribution, in which case $\vec{P}\cdot W\left(X\right)$ reaches the maximum.

This completes the proof.    □

**Property** **2.** *E(X) is an increasing function with respect to the independent variable n which represents the number of basic events.*

**Proof.**  With Equation (10), for any positive integer *k*, we have

(15) $$\begin{array}{cc} E\left(X_{k+1}\right)-E\left(X_{k}\right) & =-\sum_{i=1}^{k+1}p_{i}log_{2}p_{i}+\sum_{i=1}^{k}p_{i}log_{2}p_{i}\\ & =-p_{k+1}log_{2}p_{k+1}. \end{array}$$

As $0\leq p_{i}\leq 1$, for i=1,2,⋯,k+1, then $log_{2}p_{k+1}\leq 0$, which will lead to the fact that

(16) $$-p_{k+1}log_{2}p_{k+1}\geq 0.$$

That is $E\left(X_{k+1}\right)\geq E\left(X_{k}\right)$.

This completes the proof.    □

It can be found easily that the above properties are also true for a given graph data G=(V,E). Because in the aspect of a node’s degree, once $d_{i}$, take $v_{i}\in V$, for example, is greater than that of any $v_{j}\in V$ for i≠j, the importance of node $v_{i}$ is greater than any other node. Furthermore, a node will have greater importance if its neighbors have uniform degree distribution [28]. On these bases, in what follows, we try to use information entropy, i.e., Equation (10), to determine the node importance in a whole new perspective.

Before giving the score function to measure the node importance, at first we propose two notations, $W(v_{i})$ and $W^{+}\left(v_{i}\right)$. Take $v_{i}$ for example:

- $W(v_{i})$ represents the sum of self-information of edges with $v_{i}$ as one of its endpoint. In mathematical form, it takes the calculation form
  (17) $$W\left(v_{i}\right)=\sum_{v_{j}\in N\left(v_{i}\right)}W\left(v_{i},v_{j}\right).$$
- $W^{+}\left(v_{i}\right)$ represents the sum of self-information of edges that $v_{i}$ and its neighbors are one endpoint of these edges, and it has the following calculation formula:
  (18) $$W^{+}\left(v_{i}\right)=\sum_{v_{j}\in \Gamma \left(v_{i}\right)}W\left(v_{j}\right),$$
  where $\Gamma \left(v_{i}\right)=N\left(v_{i}\right)\cup \left\{v_{i}\right\}$.

Obviously, $W(v_{i})$ reflects the influence of edges directly connected to $v_{i}$, while $W^{+}\left(v_{i}\right)$ takes edges related to neighbors into account. Based on these discussions, the probability corresponding to any $v_{j}\in \Gamma \left(v_{i}\right)$ can be defined as

(19) $$P\left(v_{j}\right)=\frac{W(v_{j})}{W^{+}\left(v_{i}\right)}.$$

One can find that this definition satisfies the condition that the sum of probabilities is equal to 1, that is

(20) $$\sum_{v_{j}\in \Gamma \left(v_{i}\right)}\frac{W(v_{j})}{W^{+}\left(v_{i}\right)}=1.$$

To this, the information entropy of node $v_{i}$, for i=1,2,⋯,n, can be determined by the following equation:

(21) $$E\left(v_{i}\right)=-\sum_{v_{j}\in \Gamma \left(v_{i}\right)}P\left(v_{j}\right)log_{2}P\left(v_{j}\right).$$

On one hand, the information entropy can be used to quantify the amount of information contained in a random variable. On the other hand, the amount of information contained in nodes is inseparable from edges in graph data. Therefore, we can use $E(v_{i})$ which combines information entropy and edge weights as a suitable score function for each node.

**Figure 1:**
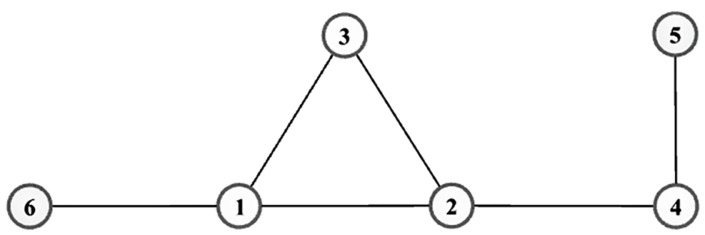
A simple graph data with n=6 and m=6.

**Example** **1.** *To make it easy to understand how to calculate the information entropy of each node, in what follows, we apply a simple graph data shown in* Figure 1 *to describe the whole process in detail.*

With Equation (8), the weight of each existing edge can be determined. The results are listed in Table 1.

Taking node $v_{1}$ for example, the value of $W(v_{1})$, $W^{+}\left(v_{1}\right)$ and $E(v_{1})$ could be obtained by Equations (17), (18) and (21), which are

(22) $$\begin{array}{cc} W(v_{1}) & =W\left(v_{1},v_{2}\right)+W\left(v_{1},v_{3}\right)+W\left(v_{1},v_{6}\right)\\ & =7.3399, \end{array}$$

(23) $$\begin{array}{cc} W^{+}\left(v_{1}\right) & =W\left(v_{1}\right)+W\left(v_{2}\right)+W\left(v_{3}\right)+W\left(v_{6}\right)\\ & =22.4348 \end{array}$$

and

(24) $$\begin{array}{cc} E(v_{1}) & =-\sum_{v_{j}\in \Gamma \left(v_{1}\right)}\frac{W(v_{j})}{W^{+}\left(v_{1}\right)}log_{2}\frac{W(v_{j})}{W^{+}\left(v_{1}\right)}\\ & =1.8161, \end{array}$$

the same as that of $v_{2},v_{3},v_{4},v_{5}$ and $v_{6}$. All in all, the information entropy for all nodes can be calculated and here we list it in Table 2.

For nodes $v_{i},v_{j}\in V$, if $E\left(v_{i}\right)\geq E\left(v_{j}\right)$, then the importance rank result can be expressed as $v_{i}≽v_{j}$, and otherwise, it can be expressed as $v_{i}≺v_{j}$ or $v_{j}≻v_{i}$. As can be seen from Table 2, one has that $E\left(v_{2}\right)>E\left(v_{1}\right)>E\left(v_{3}\right)>E\left(v_{4}\right)>E\left(v_{5}\right)>E\left(v_{6}\right)$, then these six nodes can be ranked as $v_{2}≻v_{1}≻v_{3}≻v_{4}≻v_{5}≻v_{6}$.

### 3.3. Summary of Algorithm

In this part, we give the detailed process of our proposed node importance ranking method. For convenience, in what follows, we apply SIWR to represent the proposed method. The input of the algorithm is a graph data G=(V,E) with *n* nodes and *m* edges, and its output is the possible rank result, such as $v_{i1}≽v_{i2}≽\cdots ≽v_{in}$.

The construction of Algorithm 1 is operated in the following three phases: weight computation (lines 2–4), information entropy computation (lines 5–9) and nodes ranking (lines 10–16).
**Algorithm 1:** The construction procedure of SIWR algorithm **input** : Graph data G=(V,E). **output**: Possible rank result $v_{i1}≽v_{i2}≽\cdots ≽v_{in}$.  
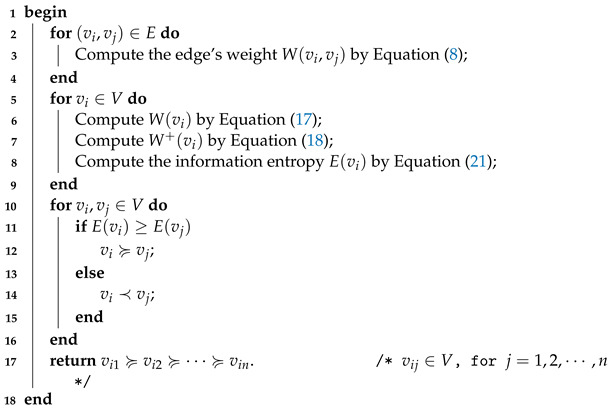



## 4. Experimental Construction

In this section, we prepare the experimental environment, such as the experimental platform, experimental datasets and evaluation criteria.

### 4.1. Experimental Platform

The algorithm development platform is the following: MATLAB 2018a. The computer configuration is the following: Intel(R)Core(TM)i5-8250U CPU and the 64-bit Windows 10 operation system. For ease of reading, the detailed information is listed in Table 3.

### 4.2. Datasets Description

In this article, we perform our experiment with the following nine real-world datasets that can be downloaded from the corresponding academic website http://konect.cc/networks/(accessed on 1 September 2022). The detailed information of related datasets is given below.

- **Karate**: The social network of friendships between 34 members of a karate club at a US university in the 1970s.
- **Dolphins**: The social network of frequent associations between 62 dolphins living in New Zealand.
- **Polbooks**: The network is made up of books about US politics published in 2004.
- **Adjnoun**: The network of co-words between adjectives and nouns commonly used in the novel “David Copperfield".
- **Football**: The network of US football games between division IA colleges.
- **Jazz**: The collaborative network between jazz musicians.
- **Netscience**: The collaborative network of scientists who have published papers in the field of network science.
- **Email**: The interactive network of emails among members of the University of Rovira.
- **Friendships**: The network contains friendships between users of the website.

The topological statistical characteristics of the above datasets are listed in Table 4. Therein, each row from left to right is the name of datasets, number of nodes *n*, number of edges *m*, average degree <*d*>, maximum degree $d_{max}$ and clustering coefficient cc.

### 4.3. Evaluation Criteria

Here, we propose three evaluation criteria to evaluate the advantage and disadvantage of node importance ranking methods, which are the monotonicity-based evaluation criterion, complementary cumulative distribution function-based evaluation criterion and susceptible–infected–recovered epidemic-model-based evaluation criterion.

#### 4.3.1. Monotonicity Based Evaluation Criterion

By taking the fact that a ranking method will be better if a few nodes are listed in the same order of consideration, the monotonicity relation [41] is applied to evaluate the discriminability of different methods, and the concrete formula is

(25) $$M\left(R\right)={\left(1-\frac{\sum_{r\in \Gamma }n_{r}{\left(n_{r}-1\right)}^{2}}{n(n-1)}\right)}^{2},$$

where *R* is the possible rank result, $n_{r}$ represents the number of nodes that have been listed in the same order of *R*, and Γ is the index that represents the number of different orders. For example, if the rank result *R* is $v_{1}≻v_{2}\approx v_{3}≻v_{4}≻v_{5}$, then $\Gamma =\left\{1,2,3,4\right\}$, in which case nodes $v_{2}$ and $v_{3}$ are listed in the same order. To this, $n_{1}=n_{3}=n_{4}=1$ and $n_{2}=2$.

Obviously, the closer the value of M(R) is to 1, the greater the monotonicity of the possible rank result [42]. When all nodes have a unique order, the value of M(R) will be 1, and the possible rank result is completely monotonic.

#### 4.3.2. Complementary Cumulative Distribution Function Based Evaluation Criterion

In addition to monotonicity, the complementary cumulative distribution function, abbreviated to CCDF for convenience, was utilized to further evaluate the ability that identify the importance of different nodes [43]. The mathematical formula of it is

(26) $$CCDF\left(r\right)=\frac{n-\sum_{i=1}^{r}n_{i}}{n}.$$

Obviously, this formula can display the distribution of nodes in different orders. Having more nodes in the same order causes the function to rapidly drop to zero, while having fewer nodes in the same order will obtain a smoother descending slope.

#### 4.3.3. Susceptible-Infected-Recovered Epidemic Model Based Evaluation Criterion

In order to assess the accuracy of SIWR method, we compare the possible rank result that generated by SIWR and other benchmark methods in terms of the susceptible–infected–recovered epidemic model, i.e., abbreviated to SIR for convenience [44,45].

Each node belongs to one of three states in SIR, which are susceptible, infected and recovered, respectively. At first, node $v_{i}\in V$ is selected as infected node, while others are in a susceptible state. After that, the infected node affects its neighbors with the infected probability β=1/(<d>−1), and then enters into a recovered state with the recovery probability r=1. It should be pointed out that the infected probability and recovery probability have various forms in different articles. Here, we choose the same form as reference [46]. Finally, the total number of infected nodes is regarded as the propagation ability of node $v_{i}$ when the whole process is finished. The stronger the propagation ability, the more important the node.

To increase accuracy, this process will be repeated hundreds of times, and the mean value will be considered the final result. Its mathematical expression is given as

(27) $$F\left(t\right)=\frac{n_{I}}{N_{ite}},$$

where $n_{I}$ represents the total number of infected nodes and $N_{ite}$ represents the number of repeated experiments.

## 5. Results Analysis

In this section, we conduct an experimental analysis of the SIWR method on nine real-world datasets. The concrete analysis includes monotonicity analysis, node distribution analysis, SIR analysis, robustness analysis and running time analysis. What is more, some comparing methods are used here to support the advantage of the SIWR method, which are the DC, MI, CC, EC, KS and IKS methods.

### 5.1. Monotonicity Analysis

By computing, the value of M(R) with respect to the benchmark methods and SIWR method are listed in Table 5. Obviously, the ranking method SIWR shows excellent performance, especially on the Karate, Jazz, Netscience, Email and Friendships datasets. The interesting fact is that the good performance of the SIWR method increases along with the increasing of *n*, the nodes number of graph data.

On the Dolphins dataset, it can be seen from Table 5 that both of the EC and SIWR methods reach the maximum value at the same time. Certainly, the good performance of SIWR is obvious, especially for the KS method, and the bigger difference between them is 0.6210. What is more, the minimum difference between SIWR and the other methods, except EC, is 0.0074. For this, we can make a guess that for big graph data, the SIWR method would show more excellent performance.

On the Polbooks dataset, one can find that the MI, EC and SIWR methods reach the maximum value at the same time, which means that these three methods can completely identify the importance of different nodes and distribute each node to the unique order. What is more, the advantage is also obvious.

Due to the scale of the Adjnoun dataset being similar to that of the Polbooks dataset, most methods obtain similar monotonicity, except the KS method. Obviously, the M(R) value of the KS method on the Adjnoun dataset is significantly larger than that of Polbooks compared to other methods. The main reason is that the maximum degree of Adjnoun is much larger than that of Polbooks. In addition, nodes with larger degree are scattered on the Adjnoun dataset.

On the Football dataset, both DC and KS methods perform poorly, especially the KS method, which obtains the minimum value 0.0003. This shows that the KS method can hardly identify the importance of different nodes on the Football dataset. The reason is related to the topological characteristics of this dataset, as we can find that the minimum degree of this dataset is 7 and the maximum degree is 12, but the average degree is as high as 10.6609. Due to most nodes having the same degree, neither DC nor KS can identify the importance of nodes commendably. In this case, the EC and SIWR methods still reach the maximum value. In terms of another perspective, it confirms the advantage of the SIWR method.

On the Email and Friendship datasets, since the scale of the dataset increases, the EC method that performs well on other datasets does not achieve good results. Obviously, it can be seen from Table 5 that the SIWR method reaches the maximum value on these two datasets.

All in all, the monotonicity values of the SIWR method are vastly superior to most methods. Videlicet, the rank result produced by SIWR method distributes a lower number of nodes to the same order. This is a very nice performance result for the node ranking, especially for the dataset with a certain property, such as the uniform degree distribution of nodes, large number of nodes with high degree, and so on.

### 5.2. Node Distribution Analysis

**Figure 2:**
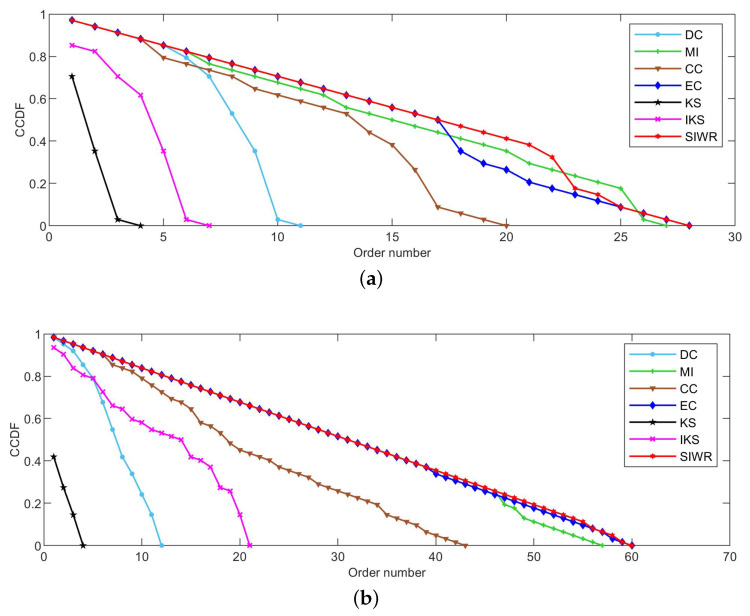
The curves of CCDF on (**a**) Karate and (**b**) Dolphins.

**Figure 3:**
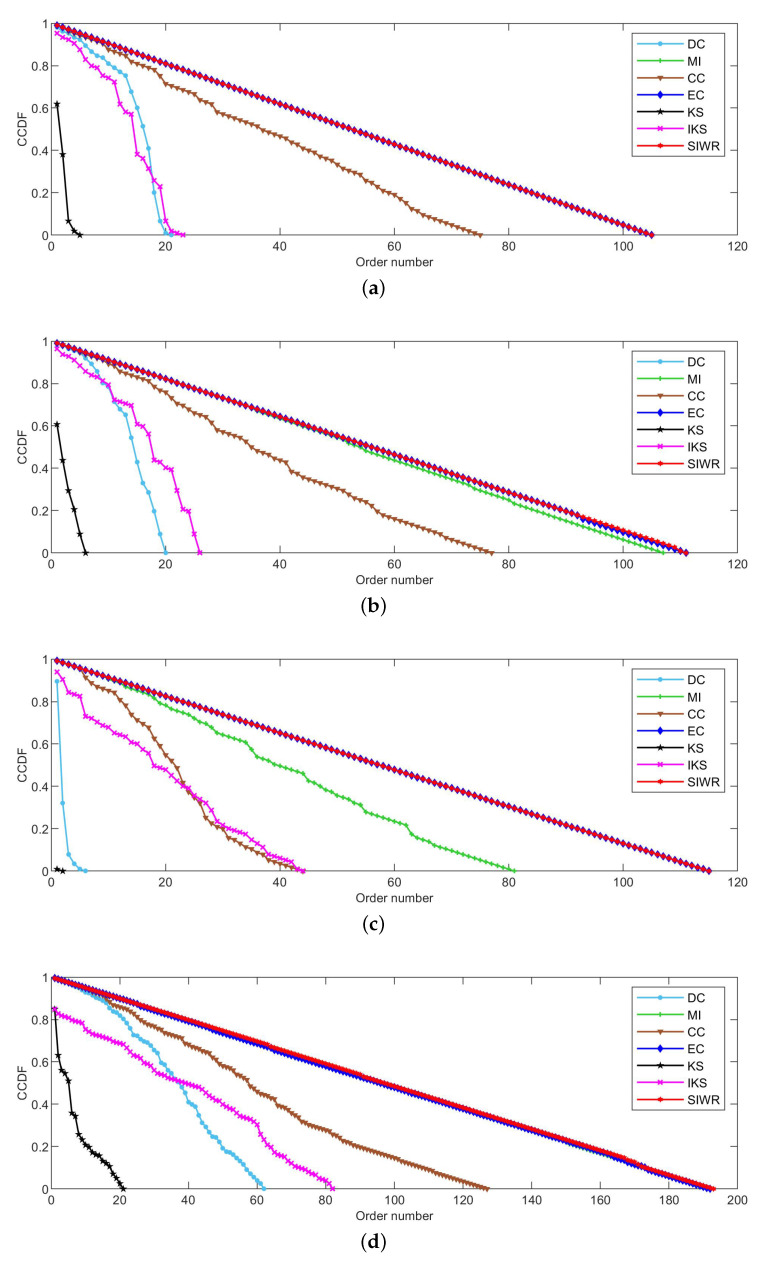

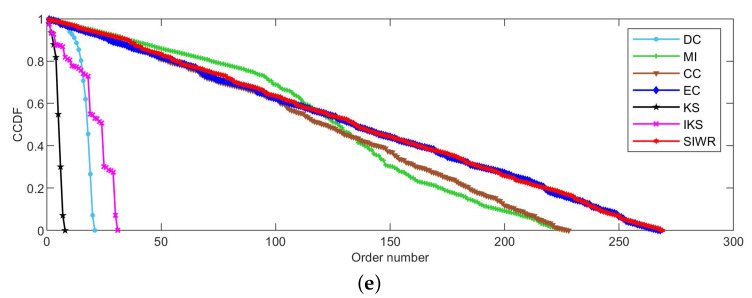
The curves of CCDF on (**a**) Polbooks, (**b**) Adjnoun, (**c**) Football, (**d**) Jazz and (**e**) Netscience.

**Figure 4:**
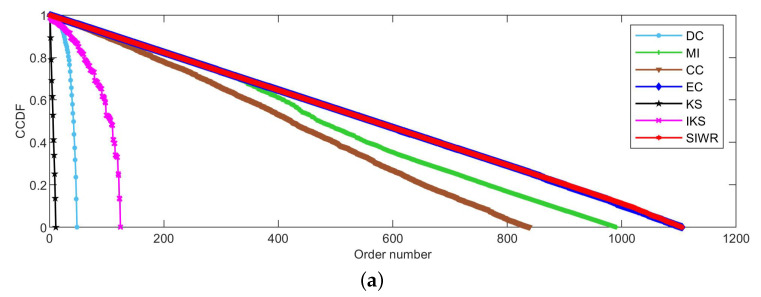

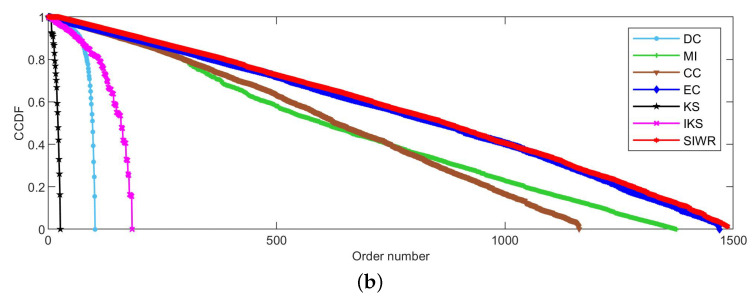
The curves of CCDF on (**a**) Email and (**b**) Friendships.

Figure 2, Figure 3 and Figure 4 reflect the curves of CCDF of DC, MI, CC, EC, KS, IKS and SIWR methods on nine datasets. Herein, the vertical axis represents the concrete value of CCDF, and the horizontal axis represents the order number in the rank result.

Figure 2a is the curve of CCDF on the Karate dataset. Obviously, there are four pentagrams on the curve induced by the KS method. The reason of it is that the index Γ is $\left\{1,2,3,4\right\}$ for it. In addition, there are as many as 10 nodes in the first order of the rank result. That is to say, the importance of these 10 nodes are equal. For a dataset with only 34 number of nodes, is it a good rank result? It is not. However, for the SIWR method, it can be easily found that the index Γ is $\left\{1,2,\cdots ,28\right\}$. To this, the descending slope of curve of CCDF with respect to the SIWR method is smoother.

As shown in Figure 2b, the SIWR method shows good ranking performance. There are 62 nodes for the Dolphins dataset, but the order number reaches 60. Frankly speaking, almost every node is located in a unique order, i.e., $n_{i}=1$ is true for i∈Γ except $n_{56}=2$ and $n_{59}=2$.

As can be seen from Figure 3a, the SIWR method can divide the Polbooks dataset into 105 sortable classes. This is a perfect rank result as the node number of this dataset is also equal to 105. The KS method still has the worst ranking ability. The CC method is neither good nor bad. More interestingly, the facts reflected in Figure 3a are consistent with those of Table 5.

In terms of the distribution of curves from left to right, as well as that of the descending slope, it is not hard to find that the Adjnoun dataset shown in Figure 3b is similar to the Polbooks dataset. However, the IKS method has a smoother descending slope than the DC method on the Adjnoun dataset. Regrettably, the curve of CCDF with respect to the IKS method descends faster at the beginning. Due to the key nodes usually being listed in the front of the rank result, the IKS method cannot better identify the key nodes. In addition, both of EC and SIWR methods obtain the highest order numbers on the Adjnoun dataset. At the same time, the advantage is also obvious.

The Football dataset contains 115 nodes, but the KS method simultaneously identifies 114 nodes as the most important nodes. This is a disastrous result. However, for the SIWR method, the order number shown in Figure 3c is 115, which is a perfect rank result. Additionally, its good rank ability is consistent with the monotonicity value of the SIWR method listed in Table 5.

As can be seen from Table 4, the edge number, average degree and maximum of the Jazz dataset are the largest among all the proposed datasets except the Email and Friendships datasets. For such datasets, the ranking methods that can make full use of edge information will have a great advantage. Based on the property of the SIWR method, one can be inferred that the descending slope of the SIWR method should be smoother, and this is verified by Figure 3d.

It can be seen from Figure 3e that the MI method obtains a smoother descending slope at the beginning. However, the slope of decline suddenly increases when the order number is between 100 and 150. The main reason is that the MI method distributes a large number of nodes with the same importance in this interval. In other words, the MI method cannot identify the importance of these nodes. On the whole, the SIWR and EC methods still show great advantages.

Obviously, Figure 4 tells us that the ranking ability of the KS, DC and IKS methods is significantly weaker than that of other methods. For the SIWR method, its overall ranking ability is quite good, as the descending slope of the CCDF curve is smooth. What is more, one can find that the value of CCDF is equal to 0, and the order number of the SIWR method comes up to 1106 in Figure 4a. In addition, the order number of SIWR method comes up to 1487, which is 15 higher than the EC method in Figure 4b. It is worth mentioning that the SIWR method obtains the maximum order number in all methods.

Based on above analysis, one can find that the curves of CCDF with respect to the SIWR method can maintain a smoother descending slope in most datasets. In other words, the SIWR method can lead to a good rank result, in which case little nodes are located at the same order.

### 5.3. SIR Analysis

In terms of SIR analysis, at first we make a rank for all nodes of each dataset by SIWR, DC, etc. After that, the nodes listed in front of the rank result are selected as seeds, and also are endowed the state of infection. Here we select 2, 4, 6, 8 and 10 nodes as seeds if n≤1000, and 10, 20, 30, 40 and 50 nodes as seeds once n>1000. What is more, the KS method is excluded from analysis because a large number of nodes have the same order number once the KS method is applied to rank it.

**Figure 5:**
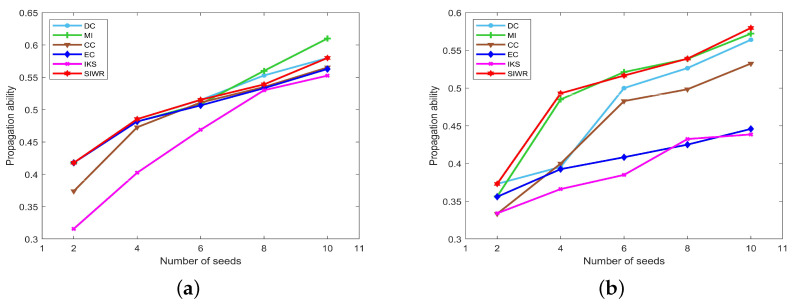
The curves of propagation ability on (**a**) Karate and (**b**) Dolphins.

**Figure 6:**
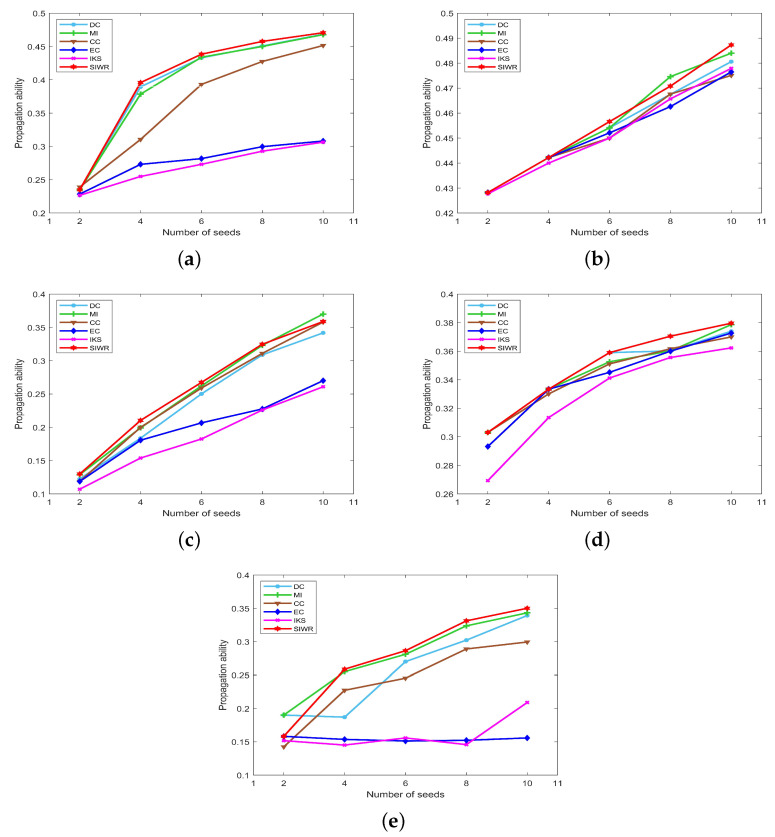
The curves of propagation ability on (**a**) Polbooks, (**b**) Adjnoun, (**c**) Football, (**d**) Jazz and (**e**) Netscience.

**Figure 7:**
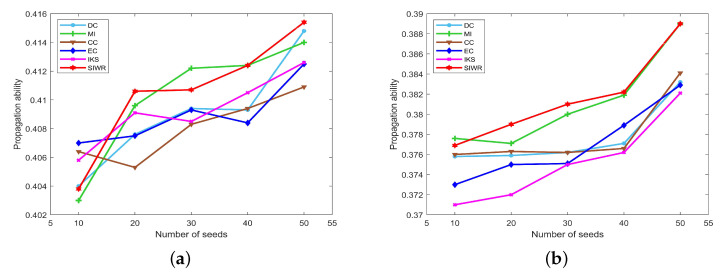
The curves of propagation ability on (**a**) Email and (**b**) Friendships.

The propagation ability of seeds obtained by DC, MI, CC, EC, IKS and SIWR methods on nine datasets is displayed in Figure 5, Figure 6 and Figure 7 wherein the horizontal axis of each subfigure represents the number of seeds and the vertical axis of each subfigure represents the propagation ability of seeds.

It can be seen from Table 4 that the Karate dataset has only 34 nodes, but the clustering coefficient is large. This indicates that the distribution of these nodes is relatively concentrated, so seeds can obtain a large propagation range on all datasets. Obviously, one can find that the maximum propagation ability is as high as 0.61 from Figure 5a. The IKS method performs the worst, and the SIWR method has the obvious advantages when the number of seeds is equal to 2, 4 and 6.

As shown in Figure 5b, the propagation ability of seeds obtained by the SIWR method is much greater than that of other methods, except for the situation that the number of seeds is equal to 6. The bigger difference between SIWR and others is up to 0.1411. It is worth mentioning that the key nodes obtained by the EC method have poor accuracy, although it performs well in both of the monotonicity and node distribution experiments.

Similar situations to the Dolphins dataset appear on the Polbooks and Football datasets. The SIWR method achieves the highest propagation ability except at a certain point, while that of EC and IKS methods is much lower compared to others. In addition, from the previous two experiments, one can find that the SIWR method distributes each node to the unique order on these two datasets. At the same time, Figure 6a,c show that the seeds with respect to the SIWR method are more influential. That is, the rank result obtained by the SIWR method not only has higher monotonicity, but also is more accurate.

From Figure 6b,d, one can find that the curves of the propagation ability on the Adjnoun and Jazz datasets are concentrated. In particular, there are multiple methods that obtain the same propagation ability when the number of seeds is equal to 2 and 4, which means that these methods obtain the same key nodes. What is more, the advantage of SIWR method is still obvious. Certainly, the SIWR method exhibits the highest propagation ability for different numbers of seeds on the Jazz dataset.

As can be seen from Figure 6e, with the increasing number of seeds, the curves of the propagation ability corresponding to the EC and IKS methods do not change much, while that of the SIWR method shows an obvious upward trend. The maximum value of the SIWR method is 0.3501, which is 0.1943 higher than the EC method and 0.1411 higher than the IKS method. This means that the number of nodes infected by the SIWR method is 73 higher than that of the EC method and 53 higher than that of the IKS method. Certainly, the advantage of the SIWR method is obvious.

From Figure 7, the curves of the propagation ability with respect to different methods are constantly fluctuating due to the scale of the dataset increasing. In this case, the SIWR method still maintains the relatively steady upward trend. Especially for the seeds with numbers of 20 or 50 nodes in Figure 7a and the seeds with number of 20 or 30 nodes in Figure 7b, the SIWR method outperforms the other methods obviously. Thus, we could deduce that the key nodes obtained by SIWR method are more accurate for large-scale graph data.

To summarize, the key nodes obtained by SIWR method show better propagation ability especially for large-scale datasets. Therefore, to some extent, the conclusion can be drawn that the SIWR method can obtain more accurate rank results and can be used in large-scale datasets.

### 5.4. Robustness Analysis

In order to analyze the robustness of the method, we randomly select nodes and remove them from the original datasets. The change rate of rank result is considered after the structure of datasets is changed. First, we randomly select 1% and 5% nodes and delete them from the original datasets. At the same time, the selected nodes are removed from the initial rank results. After that, the remaining nodes are ranked and the new rank result is obtained. Finally, we consider the proportion of nodes whose positions have changed by comparing the two rank results. The experiment will be repeated hundreds of times, and the mean value will be taken as the final change rate. Table 6 shows the rate of change after randomly removing 1% nodes from the original datasets.

The Karate and Dolphins datasets contain a smaller number of nodes. Only removing one node will not change the rank results dramatically. However, in fact, the MI, CC and EC methods do not perform well on these two datasets. In particular, the change rate of the CC method is as high as 54.79%, which means that only removing one node will cause more than half of the orders to change in the final rank results. In this case, the DC, IKS and SIWR methods are relatively stable and float between 25% and 45%.

The football datasets are the most special. This dataset has a relatively large average degree and clustering coefficient, which means that the nodes in this dataset are concentrated. The change of the local structure will affect the entire structure to a greater extent. Because of its special topological properties, the change rate of all methods is greater than 80% when only one node is removed. In this case, the SIWR method shows better robustness than the MI, CC and EC methods.

On the Email and Friendships datasets, 11 and 19 nodes were removed, respectively. The change rate of rank results obtained by all method increases significantly compared with other datasets. Obviously, the SIWR method obtains the minimum change rate even better than the DC and IKS methods.

Table 7 shows the rate of change after randomly removing 5% nodes from the original datasets. On the whole, the change rate of all methods increases significantly. The interesting phenomenon is that the advantage of the IKS method disappears and the DC method achieves the minimum rate of change on the Karate and Dolphins datasets. The rate of change obtained from the SIWR method is second only to the DC method on these two datasets.

The advantage of the SIWR method is reflected in the Netscience, Email and Friendships datasets. The structure of the dataset is changed dramatically after removing 8, 57 and 93 nodes from these three datasets, respectively. In this case, the SIWR method obtains the minimum rate of change, which is consistent with Table 6. Therefore, we can conclude that the SIWR method has strong robustness and can be used in large-scale datasets.

In general, the minimum rate of change is concentrated in the DC, IKS and SIWR methods. However, in the previous experiment, we verified that the DC and IKS methods do not perform well in terms of identifying the importance of nodes. These two methods usually distribute the same score to a large number of nodes. As a result, the importance of different nodes cannot be correctly identified. Although the rank results are not changed significantly after removing a few nodes, these two methods still are unable to accurately identify the importance of different nodes. What is more, the SIWR method showed obvious advantages in previous experiments compared with DC and IKS methods. Table 2 shows that the SIWR method can distribute a lower number of nodes to the same order in all datasets. Therefore, although a few nodes are removed, our method can still obtain the rank result with a small rate of change and high accuracy, especially for the big datasets. To summarize, our method is more robust than the MI, CC and EC methods and has greater advantages in large-scale datasets.

### 5.5. Running Time Analysis

**Figure 8:**
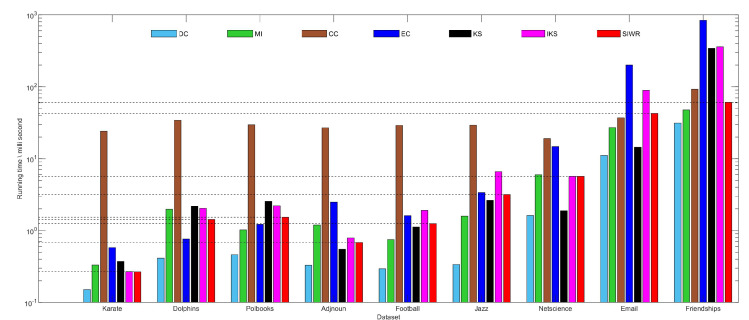
Running time of different methods on nine datasets.

As we all know, a shorter running time means that the method is faster. Figure 8 shows that SIWR method takes less time than the CC, KS and IKS methods on Karate, Dolphins, Polbooks and Friendships datasets. What is more, the running time of SIWR is also lower than that of the CC, EC and IKS methods on Adjnoun, Football, Jazz, Netscience and Friendships datasets. Since the SIWR method needs to set weight values for all edges in the graph data, the running time will increase when the number of edges is large. This is the reason that SIWR methods spend significantly more time on the Jazz dataset compared to Adjnoun and Football datasets when the number of nodes is similar.

Obviously, DC is the fastest method on all datasets. However, the rank result obtained by the DC method does not achieve better monotonicity and accuracy. The CC method needs to consider the problem of the shortest path in the graph data, so it is the slowest method on most datasets. In general, the running time of the SIWR method is in the middle position among all comparison methods.

## 6. Conclusions

This paper discussed the node importance ranking method of graph data from the perspective of edges. On one hand, the self-information that takes the nodes degree into account is regarded as the weight of edges, and it turned an unweighted graph datum into a weighted graph datum. On the other hand, we constructed the information entropy of nodes to measure the importance of each node.

A large number of theoretical derivation and experimental analyses demonstrated that the proposed method is more advantageous in aspects of monotonicity, node distribution and accuracy. However, it is not hard to see that this paper only discussed the undirected unweighted graph data. In reality, this is a special case. Therefore, a method that combines the topological properties and the theory of entropy will be considered in our future work. In addition, we will try our best to study graph data with more complicated cases, such as directed graph data, weighted graph data, and so on. 

## Figures and Tables

**Table 1:** The weight of each existing edge.

Edge	Weight
$\left(v_{1},v_{2}\right)$	3.1699
$\left(v_{1},v_{3}\right)$	2.5850
$\left(v_{1},v_{6}\right)$	1.5850
$\left(v_{2},v_{3}\right)$	2.5850
$\left(v_{2},v_{4}\right)$	2.5850
$\left(v_{4},v_{5}\right)$	1.0000

**Table 2:** The information entropy of each node.

Node	$W(v_{i})$	$W^{+}\left(v_{i}\right)$	$E(v_{i})$
$v_{1}$	7.3399	22.4348	1.8161
$v_{2}$	8.3399	24.4348	1.9309
$v_{3}$	5.1700	20.8498	1.5579
$v_{4}$	3.5850	12.9249	1.2067
$v_{5}$	1.0000	4.5850	0.7567
$v_{6}$	1.5850	8.9249	0.6748

**Table 3:** The experimental platform.

Parameter	Parameter Value
RAM	8 GB
Speed	1.8 GHz
Operation system	Windows 10
Operation programing	MATLAB R2018a
CPU	Intel(R)Core(TM)i5-8250U

**Table 4:** Topological statistical characteristics of the eight real-world graph data.

Dataset	*n*	*m*	<*d*>	$d_{\max}$	cc
Karate	34	78	4.5882	17	0.5879
Dolphins	62	159	5.1290	12	0.3030
Polbooks	105	441	8.4000	25	0.4875
Adjnoun	112	425	7.5893	49	0.1898
Football	115	613	10.6609	12	0.4032
Jazz	198	2742	27.6970	100	0.6334
Netscience	379	914	4.8232	34	0.7981
Email	1133	10903	9.6230	71	0.2550
Friendships	1858	12534	13.4919	272	0.1670

**Table 5:** The monotonicity value of seven node importance ranking methods. The best results are highlighted in bold.

Dataset	M(DC)	M(MI)	M(CC)	M(EC)	M(KS)	M(IKS)	M(SIWR)
Karate	0.7079	0.9542	0.8993	0.9576	0.4958	0.6463	**0.9577**
Dolphins	0.8312	0.9905	0.9737	**0.9979**	0.3769	0.8841	**0.9979**
Polbooks	0.8252	**1.0000**	0.9846	**1.0000**	0.4949	0.8382	**1.0000**
Adjnoun	0.8661	0.9984	0.9837	**0.9997**	0.5990	0.8745	**0.9997**
Football	0.3636	0.9835	0.9488	**1.0000**	0.0003	0.9419	**1.0000**
Jazz	0.9659	0.9993	0.9878	0.9994	0.7944	0.9383	**0.9995**
Netscience	0.7642	0.9906	0.9928	0.9952	0.6421	0.7607	**0.9954**
Email	0.8874	0.9988	0.9988	0.9995	0.8088	0.8981	**0.9999**
Friendships	0.8859	0.9977	0.9982	0.9964	0.4388	0.4996	**0.9991**

**Table 6:** The rate of change after removing 1% nodes. The best results are highlighted in bold.

Dataset	DC	MI	CC	EC	IKS	SIWR
Karate	0.2767	0.3990	0.5479	0.4898	**0.2716**	0.2953
Dolphins	0.3678	0.5370	0.5383	0.4934	**0.2827**	0.4430
Polbooks	0.3960	0.4785	0.6530	0.6810	**0.3789**	0.4454
Adjnoun	**0.3017**	0.5280	0.4510	0.3357	0.3904	0.3827
Football	**0.8359**	0.8536	0.8512	0.8884	0.8439	0.8467
Jazz	0.5123	0.6280	0.5487	0.5392	**0.3190**	0.5365
Netscience	0.7150	0.6431	0.5652	0.5876	0.6787	**0.5324**
Email	0.9081	0.8901	0.8998	0.8945	0.8431	**0.8421**
Friendships	0.9103	0.8600	0.9138	0.8622	0.8953	**0.8535**

**Table 7:** The rate of change after removing 5% nodes. The best results are highlighted in bold.

Dataset	DC	MI	CC	EC	IKS	SIWR
Karate	**0.4255**	0.5145	0.5776	0.6230	0.4770	0.4566
Dolphins	**0.6781**	0.7225	0.7135	0.7499	0.7243	0.7042
Polbooks	0.8169	0.7560	0.7862	0.8392	**0.7540**	0.8170
Adjnoun	0.7678	0.7890	0.7578	**0.7117**	0.7666	0.7531
Football	0.9170	0.9144	0.8981	0.9270	**0.8875**	0.9266
Jazz	0.7545	0.8400	0.7840	0.7428	**0.6410**	0.7537
Netscience	0.9268	0.8688	0.8585	0.8587	0.9131	**0.8519**
Email	0.9318	0.9249	0.9244	0.9360	0.9274	**0.9112**
Friendships	0.9327	0.9305	0.9254	0.9259	0.9293	**0.9150**

## Data Availability

Not applicable.
